# Variations in Antioxidant Genes and Male Infertility

**DOI:** 10.1155/2015/513196

**Published:** 2015-11-05

**Authors:** Bolan Yu, Zhaofeng Huang

**Affiliations:** ^1^Key Laboratory for Major Obstetric Diseases of Guangdong Province, Third Affiliated Hospital of Guangzhou Medical University, Guangzhou 510150, China; ^2^Key Laboratory of Reproduction and Genetics of Guangdong Higher Education Institutes, Third Affiliated Hospital of Guangzhou Medical University, Guangzhou 510150, China; ^3^Institute of Human Virology, Zhongshan School of Medicine, Sun Yat-sen University, Guangzhou 510080, China; ^4^Department of Biochemistry, Zhongshan School of Medicine, Sun Yat-sen University, Guangzhou 510080, China; ^5^Key Laboratory of Tropical Diseases Control, Sun Yat-sen University, Ministry of Education in China, Guangzhou 510080, China

## Abstract

Oxidative stress and reactive oxygen species (ROS) are generated from both endogenous and environmental resources, which in turn may cause defective spermatogenesis and male infertility. Antioxidant genes, which include catalase (*CAT*), glutathione peroxidase (*GPX*), glutathione *S*-transferase (*GST*), nitric oxide synthase (*NOS*), nuclear factor erythroid 2-related factor 2 (*NRF2*), and superoxide dismutase (*SOD*), play important roles in spermatogenesis and normal sperm function. In this review, we discuss the association between variations in major antioxidant genes and male infertility. Numerous studies have suggested that genetic disruption or functional polymorphisms in these antioxidant genes are associated with a higher risk for male infertility, which include low sperm quality, oligoasthenoteratozoospermia, oligozoospermia, and subfertility. The synergistic effects of environmental ROS and functional polymorphisms on antioxidant genes that result in male infertility have also been reported. Therefore, variants in antioxidant genes, which independently or synergistically occur with environmental ROS, affect spermatogenesis and contribute to the occurrence of male infertility. Large cohort and multiple center-based population studies to identify new antioxidant genetic variants that increase susceptibility to male infertility as well as validate its potential as genetic markers for diagnosis and risk assessment for male infertility for precise clinical approaches are warranted.

## 1. Introduction

Reactive oxygen species (ROS), which are strongly linked with oxidative stress, are oxygen-derived free radicals that include superoxide anions, hydroxyl, peroxyl, alkoxyl radicals, and hydrogen peroxide [1]. ROS can be generated either from endogenous physical processes such as mitochondrial respiration and seminal leukocytes [2] or from various environmental factors, which include drugs, pollution, toxins, smoking, radiation, and diet [3]. In sperm, ROS can cause potential damage to plasma membrane and DNA integrity, motility, and overall semen quality [2, 4, 5]; therefore, scavenging excess ROS is mandatory for normal spermatogenesis and fertilization.

The nuclear factor erythroid 2-related factor 2/antioxidant response element (NRF2/ARE) signaling pathway and its regulated antioxidant enzymes have been shown to play crucial roles in cellular oxidative stress defense during spermatogenesis and fertilization [6, 7]. Antioxidant enzymes and molecules such as superoxide dismutases (SODs), glutathione (GSH), and catalases (CATs) are largely abundant in semen plasma or in sperm cells [8–10]. Most of these genes, including* NRF2*,* SOD*,* CAT*, glutathione* S*-transferase (*GST*), glutathione peroxidase (*GPX*), and nitric oxide synthase (*NOS*), harbor sequence variants in humans, which in turn may cause male infertility in different ways. As genetic variations are an important etiological factor in male infertility, these may significantly contribute to the incidence of male infertility, especially under environmental ROS stress [11]. To date, functional polymorphisms of antioxidant genes* NRF2*,* SOD*,* GST*,* NOS*,* CAT*, and* GPX* have been reported to be associated with male infertility in humans.

This review discusses the recent progress in the study of genetic variations in antioxidant genes that have associated with male infertility. The findings of these studies indicate that functional polymorphisms in the* NRF2*,* SOD*,* GST*,* NOS*,* CAT*, and* GPX* genes may potentially contribute to genetic causes of male infertility. As the incidence of male infertility continues to increase, the analysis of its association with sequence variants in antioxidant gene may not only help understand the roles of antioxidant signaling network in ROS-related male infertility but also facilitate validating its potential as genetic markers for the diagnosis and risk assessment for male infertility in the clinic.

## 2. Antioxidant Enzymes in Spermatogenesis

**Figure 1:**
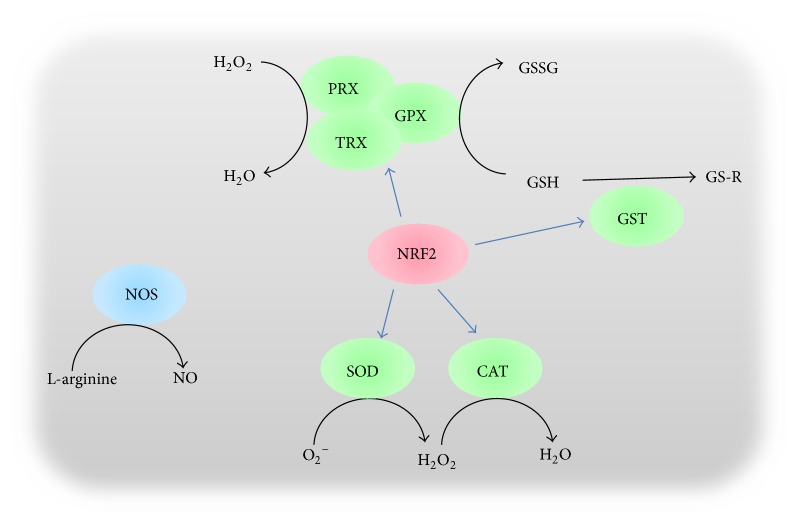
Major antioxidant gene products important for spermatogenesis. NRF2 regulates the expression of many antioxidant enzymes including peroxiredoxin (PRX), thioredoxin (TRX), glutathione peroxidase (GPX), glutathione S-transferase (GST), superoxide dismutases (SODs), and catalase (CAT). The principal form of ROS is anion superoxide (O_2_^−^), which can be converted into hydrogen peroxide (H_2_O_2_) by SODs. H_2_O_2_ can be catalyzed to H_2_O by CAT, TPX, or PRX. GST catalyzes the conjugation of the reduced glutathione (GSH) to xenobiotic substrates. Nitric oxide synthases (NOSs) catalyze the production of nitric oxide (NO) from L-arginine. GS-R, GSH-xenobiotics adducts; GSSG, oxidized glutathione.

A number of antioxidant genes involved in spermatogenesis have been identified in mammals, which include* NRF2*,* SOD*,* CAT*,* GPX*, peroxiredoxin (*PRX*), glutaredoxin (*GRX*), thioredoxin (*TRX*), and* NOS* [6, 7, 55–57]. The enzymes encoded by these genes are widely involved in the cellular antioxidant response, GSH synthesis and reduction, and thiol redox cycles during spermatogenesis or involving sperm (Figure 1). Most of these genes also contain the ARE motif in its promoter regions, which facilitates the regulation of the oxidative stress-activated NRF2 transcription pathway [58].

*NRF2* is the key gene in antioxidant defense, as it is the nuclear transcriptional factor that can induce antioxidant enzymes via ARE element [59]. In response to oxidative stress, NRF2 binds to AREs, mediating transcriptional activation of its responsive genes and modulating* in vivo* defense mechanisms against oxidative damage [60]. Kelch-like ECH-associated protein 1 (KEAP1) is the cytosolic regulatory protein of NRF2 and the sulfhydryl-rich sensor that responds to oxidants or electrophiles [61]. Under basal conditions, KEAP1 associates with NRF2 and targets it for degradation, and then modified KEAP1 by oxidative reagents will dissociate with NRF2 that could translocate into nucleus, bind to target gene ARE element, and promote many antioxidant enzyme gene expressions [62, 63].

Among the genes regulated by the NRF2-ARE signaling pathway, SODs and CATs are important enzymes that protect sperm from oxidative damage by superoxide and hydrogen peroxide (H_2_O_2_). SODs catalyze the dismutation of the superoxide radical into either ordinary molecular oxygen or hydrogen peroxide. Three families of SOD isoenzymes have been identified in humans: soluble SOD or CuZn SOD (SOD1), mitochondrial SOD or Mn SOD (SOD2), and extracellular SOD or EC SOD (SOD3) [13]. Among these, isoenzyme SOD2 is highly expressed in human semen [8, 13]. Seminal CAT catalyzes the degradation of H_2_O_2_ to oxygen and water [64], which are involved in the maintenance of normal levels of ROS and protection of spermatozoa against potentially toxic ROS [9].

NOSs are a family of enzymes that catalyze the production of nitric oxide (NO) from L-arginine [65], which is considered as an antioxidant that scavenges ROS at low concentrations [66–68]. The role of NO in sperm motility and its effect on fertility have been proven in penile erection, sperm motility and viability, metabolism, and acrosomal reaction [14]. Three NOS isoenzymes have been identified in mammals, which include neuronal NOS (nNOS; NOS1), inducible NOS (iNOS; NOS2), and endothelial NOS (eNOS; NOS3) [69].

GSTs are abundant cytosolic proteins that catalyze the conjugation of GSH to electrophilic xenobiotic substrates, which usually form ROS* in vivo *[15]. The GST family consists of three superfamilies: the cytosolic, mitochondrial, and microsomal GSTs [15, 70]. In humans, GSTs include mitochondrial GSTK1, microsomal MGST1–MGST3, and cytosolic GSTA1–GSTA5, GSTZ1, GSTM1–GSTM5, GSTO1–GSTO2, GSTP1, and GSTT1–GSTT4 [71].

TPX, PRX, and GRX are enzymes involved in the redox of thiols in cells. TRXs and GRX collaboratively catalyze the reduction of protein mixed disulfides [72–74]. TRX isoenzyme TRX1 is located in the cytosol and the nucleus, and TRX2 is exclusively expressed in the mitochondria [75, 76]. PRX enzymes are a group of highly abundant peroxidases that eliminate organic hydroperoxidase and H_2_O_2_. The glutathione peroxidase (GPX) protein family catalyzes thiol redox with glutathione [18]. Among its isoenzymes, GPX4 is predominant in the testis and is currently considered vital for spermatogenesis [52]. GPX5 is solely expressed in the caput epididymis and possibly functions in maintaining sperm DNA integrity [54].

Studies employing animal models have further confirmed that mRNAs encoding several antioxidant genes can be detected at steady-state levels in the mouse testis [77]. For example,* SOD2* mRNA levels are developmentally regulated to reach maximal levels of expression in early post-meiotic germ cells, whereas the levels of* GPX* and* CAT* mRNAs are relatively constant [77].* TPX* and* PRX* are extensively expressed in testis, and their roles in spermatogenesis have mainly been studied by gene disruption in mouse models [16, 17, 19]. In summary, antioxidant genes, including* NRF2*,* SOD*,* CAT*,* GPX*,* PRX*,* GRX*,* TRX*, and* NOS*, function at different stages of spermatogenesis, and defects in their expression may significantly contribute to the occurrence of male infertility (Table 1).

## 3. Genetic Variations in Antioxidant Genes Associated with Male Infertility

### 3.1. *NRF2*

*Nrf2* disruption has been demonstrated to affect spermatogenesis in an age-dependent manner in knockout mice model [7]. A mechanism study has shown that aged* Nrf2* knockout mice have elevated levels of lipid peroxidation in their testes and epididymis, as well as increased rates of testicular germ cell apoptosis and decreased levels of antioxidants compared to age-matched wild-type mice [7]. In humans, two SNPs (rs6721961 and rs35652124) have been associated with oligoasthenozoospermia, and individuals with 617 TT and 653 TT genotypes have a higher risk of oligoasthenozoospermia [20]. In addition, the* NRF2* rs6721961 TT genotype occurs at a higher frequency in heavy smokers with low semen quality than in those with high semen quality, and heavy smokers with this genotype have significantly lower sperm concentrations and counts compared to other genotypes [12]. At the mRNA level,* NRF2* expression was significantly lower in infertile patients than in controls [78], and a significant correlation was observed between the level of* NRF2* mRNA expression and specific sperm functional parameters such as concentration, progressive motility, immotility, and vitality [78]. Interestingly, the DJ-1 protein, which stabilizes* NRF2* by targeting 20S proteasomes in cells, has also been associated with male infertility [79–81]. The concentration of sperm DJ-1 was lower in moderate asthenozoospermia patients than in the controls [79]. Therefore, functional polymorphisms and expression level of* NRF2* as well as its regulators are associated with defective spermatogenesis in humans.

### 3.2. *GST*

Three types of* GST* SNPs, namely,* GSTT1*-null,* GSTM1*-null, and* GSTP1* Ile105Val, have been extensively demonstrated to be associated with male infertility in various ethnic populations [21–32, 34]. In a north Indian population, the* GSTT1*-null genotype was associated with nonobstructive azoospermia [21]. In Taiwanese patients with varicocele, subjects with* GSTM1*-null genotype had significantly higher 8-OHdG levels in sperm DNA and lower protein thiols and ascorbic acid in seminal plasma than those with the* GSTM1*+ genotype [22]. In a Turkish population, increased oxidative damage of sperm was higher in patients with the* GSTM1*-null genotype than in controls [23], and similar results have also been reported in Egyptian, Iranian, and Brazilian infertile patients [24–26]. In a Chinese population, the null genotype of* GSTM1* and* GSTT1* is associated with an increased susceptibility to impaired spermatogenesis such as idiopathic azoospermia or oligospermia [27–30]. The association of polymorphisms in* GSTM1*,* GSTT1*, and* GSTP1* with idiopathic azoospermia or oligospermia was also observed in a southwest Chinese population [31]. Moreover, genetic polymorphisms in* GSTT1* may also affect the surgical outcome of varicocelectomies, and the* GSTT1* genotype can affect surgical outcomes of Japanese patients such as improvement of semen parameters after varicocelectomy [32].

Meta-analysis further confirmed that* GSTM1*-null and* GSTT1*-null polymorphisms are associated with male infertility risk [82–85]. A recent analysis encompassing 6934 subjects indicated that the* GSTM1*-null genotype was significantly associated with idiopathic oligozoospermia, while the null genotype of* GSTT1* was significantly associated with normozoospermia and azoospermia, and the association between* GSTM1* polymorphism and male infertility was observed in cohorts of both Asian and Caucasian groups [84].

GST enzymes are also important in protecting sperm from cryopreservation of semen, as this process can produce large amounts of ROS. In freeze-thawed bull semen, a C/G missense mutation in rs135955605 within the* GSTM1 *gene is associated with cellular ATP content and total sperm motility [33]; therefore, genetic variations in GSTs may affect male fecundity, including sperm quality and the outcomes of semen cryopreservation.

### 3.3. *SOD*

It has long been known that seminal SOD activity is positively associated with sperm concentration and overall motility, whereas it is inversely associated with sperm DNA fragmentation [8, 38]. Genetic variations in SOD may also be related to reproductive outcomes. The Ala16Val polymorphism in the* SOD2* gene is associated with infertility and pregnancy rate in IVF cycles [39]. In a case-control study, the presence of the Ala-*MnSOD* allele (rs4880) was associated with a significant increase in the risk of infertility in male subjects [40]. Infertile men with* SOD2* rs4880 CC variants showed a low level of SOD activity [38]. In a Chinese population, the* SOD2* Val16Ala (rs4880) variant is associated with a significantly higher risk for male infertility, higher levels of sperm DNA fragmentation and 8-OHdG, and a low level of SOD activity [38, 41]. When multiple antioxidant gene variations were analyzed, the* PON1* Arg192Glu (rs662) and* SOD2* Val16Ala (rs4880) variants were associated with a significantly higher risk of male infertility and levels of sperm DNA fragmentation and 8-OHdG [41].

In rat models, it has been shown that SODs may play an important role in testicular development and spermatogenesis [86].* SOD* mRNA transcripts were identified in rat testes and their highest level was detected in tubules just prior to spermiation [86]. In a Drosophila model, null mutants for* CuZn-Sod* (SOD1) are male sterile, and the transgene of a bovine* CuZn-Sod *can rescue its male infertile phenotype [42]. In addition, an accelerated impairment of spermatogenic cells was observed in* Sod1*-knockout mice under heat stress [43]. Therefore, genetic disruption or functional polymorphisms in both SOD1 and SOD2 can lead to defective spermatogenesis.

### 3.4. *NOS*

In the testis, eNOS is responsible for NO synthesis during spermatogenesis, and genetic variants of* eNOS* may be potential risk factors for impaired spermatogenesis [45]. Several* eNOS *alleles have been associated with sperm defects in various ethnic populations. In Egyptian infertile oligoasthenoteratozoospermic men, a significant relationship between* eNOS *polymorphisms T786C and G894T with decreased sperm parameters and increased seminal oxidative stress was observed [46]. In an Italian population, the* eNOS* 894G>T variant was associated with asthenozoospermia and sperm motility [48]. Similar results were reported in a Chinese cohort [49]. In Korean infertile men, sperm morphology was associated with the 4a4b eNOS polymorphism, a sequence variant with variable number of tandem 4a4b repeats in intron 4 [51]. In Iranian males,* eNOS* “-786C,” “894T,” and “a” alleles were associated with an increased risk for poor semen parameters [47]. In a Chinese case-control study, the* eNOS* rs1799983 polymorphism was positively associated with higher levels of sperm DNA fragmentation and an increased risk for male infertility [50]. Another study involving a Chinese population showed that four common polymorphism loci, namely,* eNOS* alleles -786C of T-786C and 4A of 4A4B, as well as genotype TC of T-786C and AB of 4A4B, were significantly associated with idiopathic male infertility [45]. Taken together, these studies demonstrate that genetic variations in* eNOS* are a risk factor for decreased sperm quality, including DNA fragmentation, sperm motility, and seminal ROS.

### 3.5. *GPX*

There are three isoforms of GPX, namely, cytosolic, mitochondrial, and nuclear GPX [87]. In a mouse model, cytosolic GPX4 was essential for embryonic development and spermatogenesis [53], and the deletion of mitochondrial GPX4 (mGPX4) also caused male infertility, which in turn led to impaired sperm quality and severe structural abnormalities, reduced sperm motility, and mitochondrial membrane potential [52, 88]. In bulls, subjects with the* ETFA* TT genotype presented the highest GPX activity in cryopreserved sperm [89]. In humans, GPX-defective spermatozoa were observed in 26% of infertile men diagnosed with oligoasthenozoospermia [90]. Another study has suggested that the expression of phospholipid hydroperoxide glutathione peroxidase (PHGPx) protein, a selenoprotein belonging to the family of glutathione peroxidases, may be associated with oligoasthenozoospermia; however, no* GPX* polymorphism has been associated with male infertility to date [91]. Further examination of* GPX4* polymorphisms as a potential cause of infertility is thus warranted.

### 3.6. *CAT*

Catalase enzyme activity (CAT) was demonstrated to be associated with low sperm quality [44, 92], and one study reported that* CAT*-262T/T genotype was negatively associated with infertility in idiopathic infertile males [44].

## 4. Interaction of Antioxidant Genetic Variations and the Environment in relation to Male Infertility

Environment and genetic variation could synergistically affect male fertility. In a Danish twin study, both genetic background and environmental factors were associated with sperm quality, sex hormone levels, and sperm chromatin stability, in which heritability accounted for ≥20% of the observed variations in sperm density, hormone level, sperm morphology, and sperm chromatin parameters, whereas the rest of the variations in sperm quality were likely due to environmental factors [93].

Several studies have demonstrated that environment and antioxidant genes can affect male infertility. In terms of occupational exposure to PAHs, subjects harboring the* GSTM1*-null genotype showed significantly higher levels of PAH-DNA adducts in sperm [35]. In Russian men, the combination of* GSTM1*,* GSTT1*, and* GSTP1* gene polymorphisms and cigarette smoking was associated with a higher risk for idiopathic infertility [36]. Our study also demonstrated that heavy smokers with* NRF2* genetic variants had a higher risk of developing low semen quality compared to other genotypes [12].

Cytochrome P450 (CYP) families may contribute to the occurrence of endogenous oxidative stress* in vivo* because these are detoxification enzymes that interact with a wide range of environmental toxins and carcinogens that can form ROS. A previous study has shown a significant synergism between* GSTM1* and* CYP1A1* genotypes and infertility among human subjects [37]. A subject carrying the variants* CYP1A1* Val/Val or* CYP1A1* Ile/Val in association with* GSTM*-null genotype has a 6.90-fold higher risk for infertility than a subject carrying* CYP1A1* Ile/Ile in association with a* GSTM1* wild-type genotype [37]. Therefore, genetic polymorphisms of xenobiotic-metabolizing enzymes may also interact with antioxidant genes for environment-induced infertility [37].

## 5. GWAS Study in Male Infertility

With the development of new genetic analysis approaches, genome-wide association study (GWAS) has been utilized for male infertility recently, and new loci for male infertility have been identified using GWAS. In a large cohort of men of European descent, 172 candidate polymorphisms for association with oligozoospermia or azoospermia were evaluated and several SNPs were identified or confirmed to be significantly associated with oligozoospermia and/or azoospermia [94]. Another GWAS report identified candidate genes for male fertility traits and 9 SNPs found to be associated with reduced fertility [95]. In 2011 and 2014, two large scale GWAS in Chinese populations first discovered some new loci for the risk of nonobstructive azoospermia (NOA). A three-stage GWAS of 2,927 individuals with NOA and 5,734 controls identified significant associations between NOA risk and common variants near* PRMT6* (rs12097821),* PEX10* (rs2477686), and* SOX5* (rs10842262) [96]. A later extended three-stage validation study using 3,608 NOA cases and 5,909 controls further identified additional risk loci, including a new related gene* GEK* (Genghis Khan, orthologous to human CDC42BPA) which can cause severe male fertility in a Drosophila model [97].

A detailed summary of GWAS in infertile men has been described by Aston [98], which is not the focus of this review. However, except for the identification of new loci for male infertility, GWAS do confirm the association between previously identified SNPs in antioxidant genes and male infertility. For instance, in recent GWAS on genetic makers for sperm quality in bulls [99–101], the antioxidant genes* GSTT1*,* GSTM1*, and* NOS3* were identified as significant markers or suspected of being significantly associated with bull sperm concentration [100].

However, antioxidant signaling pathways involved in male infertility have not been analyzed at the genome-wide level to date. In addition, only a few diseases such as azoospermia or oligozoospermia have been studied at the genome-wide level. The most common male infertility disorders such as asthenozoospermia and oligoasthenozoospermia have not been extensively studied to date. Therefore, using advanced genetic analysis technologies to study antioxidant genetic variations in relation to male infertility at a genome-wide level is imperative.

## 6. Conclusions

As environmental pollution and lifestyle changes are prevalent in the current society, ROS from pollution, radiation, high-fat diets, and sedentary, physically inactive lifestyles will likely contribute to the increase in incidence of male infertility. The antioxidant enzyme system, which is largely regulated by the NRF2-ARE system, may be one of the key components that play a protective role against ROS damage during spermatogenesis and for sperm function (Figure 1). Therefore, it is expected that genetic variations in major antioxidant genes will alter the susceptibility of a male to infertility and defective spermatogenesis.

In the past two decades, numerous studies have demonstrated that functional polymorphisms or the genetic disruption of the* CAT*,* GPX*,* GST*,* NOS*,* NRF2*, and* SOD* genes was associated with male infertility (Table 2). In animal models, knocking out* Nrf2*,* Sod*, and* Gpx *all leads to mild or severe male infertility. Previous studies involving various ethnicities in different geographical regions and countries have described the association between SNPs in the* CAT*,* GPX*,* GST*,* NOS*,* NRF2*, and* SOD* genes and infertility. Several studies have also reported the synergistic effects of antioxidant gene polymorphisms and environmental ROS such as smoking and PAH exposure. Therefore, antioxidant-related genes may play a crucial role in spermatogenesis and sperm function, and their genetic variations may modify the antioxidant capability of the human reproductive system and increase the risk for male infertility.

However, most studies of the association between antioxidant gene variations and male infertility have been conducted in animal models or in a specific geographical population. In addition, systematic studies of the complete antioxidant signaling pathways in spermatogenesis and studies in multiple centers or large cohort studies are limited. Furthermore, epigenetic alterations in antioxidant genes, which may change their transcriptional activity* in vivo*, have not been examined to date. New technologies such as next-generation sequencing can yield large amounts of information at the genome-level. Therefore, the discovery and validation of antioxidant genetic variants as genetic markers for the diagnosis and risk estimation for male infertility may facilitate the improvement of clinical approaches for this particular disorder.

## Figures and Tables

**Table 1:** Major antioxidant enzymes in spermatogenesis.

Enzyme	Name	Isoforms in human	Reference
NRF2	Nuclear factor erythroid 2-related factor 2	NRF2	[12]
SOD	Superoxide dismutase	SOD1, SOD2, SOD3	[13]
CAT	Catalase	CAT	[9]
NOS	Nitric oxide (NO) synthase	NOS-1, NOS-2, NOS-3	[14]
GST	Glutathione S-transferase	GSTA1–GSTA5, GSTZ1, GSTM1–GSTM5, GSTO1-GSTO2, GSTP1, GSTT1–GSTT4	[15]
PRX	Peroxiredoxin	PRX1–PRX6	[16, 17]
GPX	Glutathione peroxidase	GPX1–GPX8	[18]
TRX	Thioredoxin	TRX1, TXR2	[19]

**Table 2:** Reported antioxidant genetic variations associated with male infertility.

Gene	Variation	Official description	Trait/effect	Species	Reference
*NRF2*	Deletion	—	Subfertility	Mice	[7]
	rs6721961 G>T	NC_000002.11:g.178130037 T>G	Oligoasthenozoospermia	Human	[20]
	rs35652124T>C	NC_000002.11:g.178130073 T>C	Oligoasthenozoospermia	Human	[20]
	rs6721961 G>T + smoking	NC_000002.11:g.178130037 T>G	Sperm concentration and count	Human	[12]

*GST*	GSTM1-null	Deletion	Male infertility, oligozoospermia, male infertility with varicocele	Human	[21–28]
	GSTT1-null	Deletion	Male infertility, male infertility with varicocele	Human	[24, 26–32]
	GSTM1 rs135955605 C/G	—	Sperm motility after cryopreservation	Bulls	[33]
	GSTP1 (Ile105Val)	NC_000011.10:g.67585218 A>G	Male infertility, oligospermia, oligoasthenoteratozoospermia, azoospermia	Human	[26, 31, 34]
	GSTP1 (Ala114Val)	NC_000011.10:g.67586108 C>T	Male infertility with varicocele, oligoasthenoteratozoospermia	Human	[27, 34]
	GSTM1-null + PAH exposure	Deletion	PAH-DNA adducts	Human	[35]
	GSTM1-null/GSTT1-null + smoking	Deletion	Idiopathic male infertility	Human	[36]
	GSTM1-null + CYP1A1	Deletion	Male infertility	Human	[37]

*SOD*	SOD2 rs4880 CC	NC_000006.11:g.160113872 A>G	Idiopathic infertility, male infertility, pregnancy rates in IVF, sperm concentration, sperm motility, and sperm DNA fragmentation	Human	[38–41]
	SOD1 knockout	—	Male infertility	Drosophila	[42]
	SOD1 knockout	—	Spermatogenic cell damage during heat stress	Mice	[43]

*CAT*	C-262T	NC_000011.10:g.34438684 C>T	Idiopathic male infertility	Human	[44]

*NOS*	eNOS T786C	NC_000007.13:g.150690079 C>T	Male infertility, oligoasthenoteratozoospermia, idiopathic male infertility	Human	[45–47]
	eNOS G894T	NC_000007.13:g.150696111 T>G, NP_001153582.1:p.Asp298Glu,	Oligoasthenoteratozoospermia, asthenozoospermia, idiopathic male infertility	Human	[46–50]
	eNOS 4a/b	NC_000007.14:g.150997188_150997214-AGGGGTGAGGAAGTCTAGACCTGCTGC(2)(3)	Idiopathic male infertility, sperm morphology	Human	[45, 47, 51]

*GPX*	GPX4 deletion	—	Male infertility, sperm chromatin condensation	Mice	[52, 53]
	GPX5 deletion	—	Sperm DNA integrity	Mice	[54]
